# Facile preparation, characterization, and investigation of mechanical strength of Starchy NaCl-binder as a lightweight construction material

**DOI:** 10.1038/s41598-023-46536-8

**Published:** 2023-11-03

**Authors:** Ahmad Fahmi, Sohrab Rafati Zavaragh, Mohammad Reza Hanafi, Hamed Rahimpour, Sahar Zinatloo-Ajabshir, Ali Asghari

**Affiliations:** 1 Department of Civil Engineering, University of Bonab, Bonab, East Azerbaijan Iran; 2 Department of Civil Engineering, Amirkabir University of Technology, Tehran, Tehran, Iran; 3 Department of Civil Engineering, University of Tabriz, Tabriz, East Azerbaijan Iran; 4 Department of Chemical Engineering, University of Bonab, P.O. Box 5551395133, Bonab, Iran; 5 University of Bonab, Bonab, East Azerbaijan Iran

**Keywords:** Engineering, Materials science

## Abstract

Sodium chloride (NaCl), commonly known as salt, is a substance that is utilized in a variety of businesses, including the tourism and construction industries. Therefore, the main purpose of this article is to accommodate a salt-based building material called NaCl-binder for tourist and industrial applications. By utilizing salt mortar with varying grain sizes, food-grade corn starch as an exclusive binder agent (without using any non-starch binder), and water under microwave-cured conditions, environmentally friendly hydrophobic hybrid NaCl-binder samples with low bulk density were successfully produced. The fabrication of these samples involved an inventive utilization of small quantities of starch. This study evaluated the impact of microwave exposure time on the strength of salt samples, particle interconnectivity and chemical composition using SEM, XRD, and XRF analyses. The compressive strength of the samples showed a remarkable increase, with a 600% improvement when using 0 to 1% corn starch, and a 137% increment when using 1 to 10% corn starch, indicating a lower rate of increment with higher starch consumption. A key aspect of this research is the significant reduction in starch consumption compared to other corn starch-based materials during the manufacturing process of the incorporated materials, highlighting its novelty and importance.

## Introduction

Salt therapy is the use of small particles of salt aerosols and mineral compounds to cure respiratory disorders, skin discomforts, and joint-bone problems, which has been employed in European salt mines since the nineteenth century. This therapy approach is non-invasive and physical, does not require medicines, and is safe. This method has a positive impact on therapy and drug reduction^1^. Because of this, research indicates that salt therapy significantly boosts tourism growth^2,3^.

Nowadays, the construction of tourist complexes and villages has a high potential to attract domestic and foreign tourists and it is possible to use the potential of the salt deposited on the beaches to build complexes, parks (and even salt villages) respecting scientific and ecological principles^4^. As the Egyptians did in the twelfth century, they used blocks of salt from the Salt Lake to construct buildings, although such constructions are still carried out by old masons^5^. The world is full of examples of these endeavors. It can be mentioned as an example of a salt restaurant that was built in Shiraz by Emtiaz Architectural Group using salt materials and a kind of natural resin, and its salt is provided from a salt lake near the city^6^. Palacio de Sal, a unique salt hotel in Bolivia, utilizes blocks mined from the salt lake, annually attracts foreign tourists and generates substantial income for the hotel investors^7^. In Qatar, the plan of using salt obtained from the desalination of seawater using solar energy has been studied for different constructions^8^. Technological projects like 3D printing using salt materials have also been studied by researchers^9–11^. A type of sustainable and innovative building material for use in hot, arid climates called SaltBlock has been introduced, which uses raw materials such as ethylene–vinyl acetate polymer, salt powder, and fine sand to produce lightweight blocks^11^.

Nowadays, caves and salt-therapy complexes, as well as mud-therapy complexes, attract tourists from all parts of the world and even some modern swimming pools are equipped with salt rooms for halotherapy^6,12–17^. In the production of bricks and natural salt materials, the grinding of salt stones, which are extracted from the bottom of salt lakes or salt mines, could be used. Due to the brittleness of salt rock, the production of salt materials with a regular geometric shape is very expensive. Thus, by using salt particles as a mortar component it can be converted into salt building materials by in situ molding, pressing, or 3D printing^8–10,18^. In the phase of laboratory studies related to the Qatar salt project plan, salt powder, maltodextrin, isopropyl alcohol, water, and microwave processing were used to prepare salt samples^6,19^. The current research aims to find cost-effective alternatives for producing natural salt samples. This involves substituting high-cost maltodextrin with low-cost corn starch (starch gel) and eliminating the use of isopropyl alcohol. The goal is to make the production process more affordable and environmentally friendly. In the past decades, the production of biopolymers and the development of biodegradable and bioplastic materials from renewable sources such as starch and cellulose products have been the focus of research, and the use of these materials instead of synthetic polymers from non-renewable sources such as oil has been of great importance to reduce the environmental impact^20–24^.

In recent years, researchers at Delft University of Technology have introduced a new biodegradable building material called CoRncrete, made by curing corn starch mortar with sand and water in a microwave. The production of these innovative and environmentally friendly materials is economically viable and has acceptable mechanical strength compared to ordinary bricks^25^. The adhesion mechanism of sand particles in this building material is caused by the gelatinization of starch particles, which binds coarse and fine sand particles together, forming a composite structure with the ability to harden quickly. Also, in further complementary research, the construction of sand-starch materials by microwave curing was further investigated and the use of conventional and natural coating materials for waterproofing the samples was also studied in detail^26^. Based on the results of these investigations, various factors influence the compressive strength of starch-based building products. Among these factors, the water content, the size distribution and composition of the grains, the temperature and duration of the hardening, the starch gelatinization process, and the binding of the starch matrix with sand grains can be mentioned^25,26^.

The common point of the starchy NaCl-binder material presented in the current study with the investigations carried out at CoRncrete^25^ and starch-sandstone^26^ is the use of corn starch to produce environmentally friendly building materials without using non-ecofriendly types of cement such as Portland cement. Starch is a suitable natural polymer for the production of biodegradable materials and building materials due to its advantages such as renewability, abundance, low cost, and biodegradability. The wide use of these materials as adhesives to improve the mechanical, rheological, thermal, insulating, and other physical properties of various materials such as cement, concrete, asphalt, mortar, etc. has been of interest to researchers^27–31^. For example, starch is used as an adhesive in thermal insulation composites^30,32–36^, as an additive to modify the viscosity of concrete^37–43^, as an asphalt modifier^44^, and as a retarder in cement^45^. It has also been reported that the use of 1% corn starch in the concrete composition increases the compressive strength of the samples^46^.

The current research investigated the fabrication of NaCl-binder samples for the first time and in an innovative way using salt mortar with salt particles, and a small amount of corn starch and water under microwave curing condition. In which compared to CoRncrete, salt particles were used instead of sand particles in the manufacture of the samples. In general, corn starch at 0, 1, 5, and 10% by weight was used to prepare the samples. Corn starch in an amount of 1% (wt.) was mainly used in the form of a gel due to the proper distribution of the starch between the particles. Various parameters such as the granulation of the salt particles, curing time, and the type of starch were examined in relation to the mechanical strength (compressive and flexural strength), microstructure, and hydrophobic characteristics of NaCl-binder samples. Additionally, these samples were the first to successfully address the issue of samples' water solubility by hybrid paraffin wax and beeswax coating. The difference in the behavior of salt and sand particles, which leads to the possibility of significantly reducing starch consumption in the manufacture of introduced material compared to CoRncrete, is one of the important points of this research. Subsequently, the hydrophobicity of the samples was also examined with beeswax and a paraffin coating. In this study, the introduced NaCl-binder materials composed of salt particles (powder or aggregates) and corn starch (gel containing starch and water) can be used to produce lightweight salt materials without the need for pressure in the preparation stage (only by impact or vibration to remove excess air from the mortar).

## Materials and methods

### Fresh NaCl-binder preparation method

In the sample preparation, the waste salt as one of the raw materials was passed through the standard sieves, and the salt particles with four different sizes were obtained (according to Table 1 and Fig. 6).

**Table 1:** Salt particles used to prepare NaCl-binder samples with and without starch.

Name of salt particles	Particle diameter (μm)	Description
S1	600–1180	Pass through the #16 sieve and remain on the #30 sieve
S2	300–600	Pass through the #30 sieve and remain on the #50 sieve
S3	150–300	Pass through the #50 sieve and remain on the #100 sieve
S4	< 150	Pass through the #100 sieve and remain on the Pan

In continue, Glucosan edible corn starch was used to prepare NaCl-binder samples, in which corn starch has been used in three forms: starch powder, starch suspension, and gelatinized starch to make salt mortar. First, the starch powder was mixed with salt particles, and then water was added. Secondly, the starch was added to the salt particles after stirring for 5 min at laboratory temperature. In this phase, the starch particles are insoluble in cold water (laboratory temperature), and not only a gelatinous state does not form, but also the starch particles become suspended in water. Thirdly, starch was added to water and converted to gelatinous starch by heating on a magnetic hot plate stirrer, and then salt particles were added. In all phases, tap water was used. When starch granules are heated in the presence of water, they swell and a phase transition occurs. With enough water, this transformation, known as gelatinization, causes the starch particles to burst and dissolve in water^47,48^. Gelatinization is an irreversible process that includes granular swelling, crystalline melting, and molecular dissolution^49^. In this research, 20 g of corn starch was mixed with 315 g of water to make gelatinized starch according to an innovative method developed specifically for this research. For this purpose, 315 g of water was first poured into the Erlenmeyer flask, and 20 g of corn starch was added, and the magnet was also placed in the Erlenmeyer flask. Then, the Erlenmeyer flask containing starch and water particles along with the magnet was placed on the magnetic hot plate stirrer, and the contents of the Erlenmeyer flask were stirred for 20 min at a speed of 350 rpm (revolutions per minute) at a temperature of 100 °C. Then, by increasing the speed of the stirrer to 150 rpm, the contents inside the Erlenmeyer flask were stirred for a further 10 min until the color of the resulting gel changed from white to milky. To increase the viscosity, gelatinized starch was stirred for an additional 5 min at 150–350 rpm at 150 °C, resulting in a whiter and more viscous gel. In the end, to cool the gel, the heat source of the stirrer (hot plate) was turned off, and the contents of the flask were stirred for a further 3 min at a speed of 150 rpm at room temperature. In the end, after measuring the weight, it was found that with the evaporation of water, the weight of the produced gel decreased by 300 g (280 g water & 20 g corn starch).

### Preparation of hardened NaCl-binder samples

The significance of the current research lies in the use of starch in a very low weight (1% wt.) to produce NaCl-binder samples with good mechanical strength. Low starch consumption in the production of cost-effective salt-building materials is very important from an economic point of view. In addition, samples (without starch) were prepared as controls to evaluate the effect of not using 1% starch. Samples containing 5 and 10% starch were also prepared to compare the effect of changing the weight of starch used in the materials. The mixture design of cubic and prismatic NaCl-binder samples (without hydrophobic coating) for compressive strength and flexural strength evaluation is according to Table 2. The dimensions of the cubic and the prismatic samples are 5 × 5 × 5 cm and 16 × 4 × 4 cm, respectively. Three samples were prepared from each row of the mix design. As detailed below, two types of microwave treatments were used to heat the samples. A power level of 900 W was used in the microwave (Black + Decker 30 L, MZ30PGSS). According to a study by Kulshreshtha et al., the burning temperature of corn starch is 290 °C^25^. Therefore, the treatment time of the samples in the microwave is of considerable importance. As will be mentioned in the results, during the tests, it was observed that the curing time has a significant impact on the mechanical strength of the samples. Therefore, the effect of changing this parameter in the samples prepared with S2 salt was studied in detail. For all samples made from gelatinized starch, the weight of starch and water in the starch gel is reported separately.

**Table 2:** Mix design* of NaCl-binder.

No.	Mold	NaCl	Starch Form	Materials (g)			Curing Time (min)	Starch to Salt (wt.%)
				Water	Corn Starch	Salt		
1	Cubic	S2	Gel	28	2	200	2	1
2	Cubic	S2	Gel	28	2	200	4	1
3	Cubic	S2	Gel	28	2	200	6	1
4	Cubic	S2	Gel	28	2	200	7	1
5	Cubic	S2	Gel	28	2	200	7.5	1
6	Cubic	S2	Gel	28	2	200	8	1
7	Cubic	S2	Gel	28	2	200	10	1
8	Cubic	S2	Gel	28	2	200	12	1
9	Cubic	S2	Gel	28	2	200	14	1
10	Cubic	S2	Gel	28	2	200	16	1
11	Cubic	S2	–	30	0	200	2	0
12	Cubic	S2	–	30	0	200	4	0
13	Cubic	S2	–	30	0	200	6	0
14	Cubic	S2	–	30	0	200	7	0
15	Cubic	S2	–	30	0	200	7.5	0
16	Cubic	S2	–	30	0	200	8	0
17	Cubic	S2	–	30	0	200	10	0
18	Cubic	S2	–	30	0	200	12	0
19	Cubic	S2	–	30	0	200	14	0
20	Cubic	S2	–	30	0	200	16	0
21	Cubic	S2	Gel	28	2	200	7	1
22	Cubic	S2	Suspension	28	2	200	7	1
23	Cubic	S2	Powder	28	2	200	7	1
24	Cubic	S1	Gel	28	2	200	7	1
25	Cubic	S2	Gel	28	2	200	7	1
26	Cubic	S3	Gel	28	2	200	7	1
27	Cubic	S4	Gel	28	2	200	7	1
28	Cubic	S1	–	28	2	200	7	1
29	Cubic	S2	–	28	2	200	7	1
30	Cubic	S3	–	28	2	200	7	1
31	Cubic	S4	–	28	2	200	7	1
32	Prismatic	S1	Gel	57.4	4.1	410	7	1
33	Prismatic	S2	Gel	57.4	4.1	410	7	1
34	Prismatic	S3	Gel	57.4	4.1	410	7	1
35	Prismatic	S4	Gel	57.4	4.1	410	7	1
36	Prismatic	S1	–	61.5	0	410	7	0
37	Prismatic	S2	–	61.5	0	410	7	0
38	Prismatic	S3	–	61.5	0	410	7	0
39	Prismatic	S4	–	61.5	0	410	7	0
40	Cubic	S2	Powder	25	10	200	7	5
41	Cubic	S2	Powder	20	20	200	7	10

*Rows 1–10 are used to prepare samples from both Type 1 and Type 2 salts, while rows 11–41 are solely used to prepare Type 1 salt samples.

According to lines 1 to 10 of Table 2, triplicate cubic samples were prepared in 10 series using 1% starch gel, and according to lines 11–20, 10 samples were prepared without any use of starch therein to compare the effect of using 1% starch on the mechanical strength of the samples. According to lines 21–23, three sets of triple cubic samples were prepared using three types of 1% starch in gel, powder, and suspension states to evaluate the effect of starch use on compressive strength. Next, as shown in lines 24–31, eight sets of triplicate cubic samples with 1% and without starch were prepared using salt particles of different granulations to evaluate the effect of variations in the size of the salt particle on the compressive strength of the samples. According to lines 32–39, eight sets of triplicate prismatic samples containing 1% starch and without starch were made to evaluate the flexural strength of the samples. Finally, two sets of cubic samples containing 5 and 10% starch (in powder form) were also prepared to evaluate the effect of increased starch consumption on compressive strength.

To prepare NaCl-binder samples, a uniform salt mortar was prepared by mixing salt particles with starch gel or starch powder and water for 5 min at 50 rpm using an electric mixer (Sepehr Arya Azma-LM3). The weight ratio of starch to salt in all samples containing starch (powder or gel) equaled 1%. To produce NaCl-binder cube samples, three layers of the prepared salt mortar were poured into 5 × 5 × 5 cube molds (heat-resistant Plexiglas material) and cured in the microwave. At this stage, after pouring each layer of salt, each layer was tamped 10 times with a silicone rubber-capped steel rod^25^. It should be noted that due to the importance of the color of the samples, any oiling of the molds was avoided in this study. To prevent the salt mortar from sticking to the mold, to keep the samples intact, and to remove them safely from the mold, a binding cover was installed into the mold, allowing the NaCl-binder samples to be easily pulled out of the mold.

A 16 × 4 × 4 aluminum mold was used to make the prismatic samples, and a cellophane layer was used inside the mold to prevent the salt mortar from sticking to the aluminum mold. Salt mortar was poured into a prismatic mold in three layers and pounded. Then, using a cellophane layer, the samples were removed from the mold and transferred to the microwave (it was not possible to put the metal mold in the microwave). Three cubic samples or one prismatic sample from each test design were placed in a microwave with a power level of 900 W and processed for a specified time according to Table 2. Since the volume of all three cubic samples is 375 cm^3^ and the volume of one prismatic sample is 256 cm^3^, to make similar samples in terms of energy absorption in the microwave; a 125 cm^3^ cubic sample was placed next to each prismatic sample to make the volume reach 381 cm^3^ (only slightly more than the 375 cm^3^ volume of the three cubic samples). Due to the lower weight of the starch used in the samples, the samples did not swell after microwave curing and therefore, overhead loading of the samples was not required. After removing the samples from the microwave and lowering their temperature to laboratory temperature (25 °C), the dimensions and mass of the samples were measured to calculate the bulk density, and then their compressive and flexural strengths were also measured.

**Figure 1:**
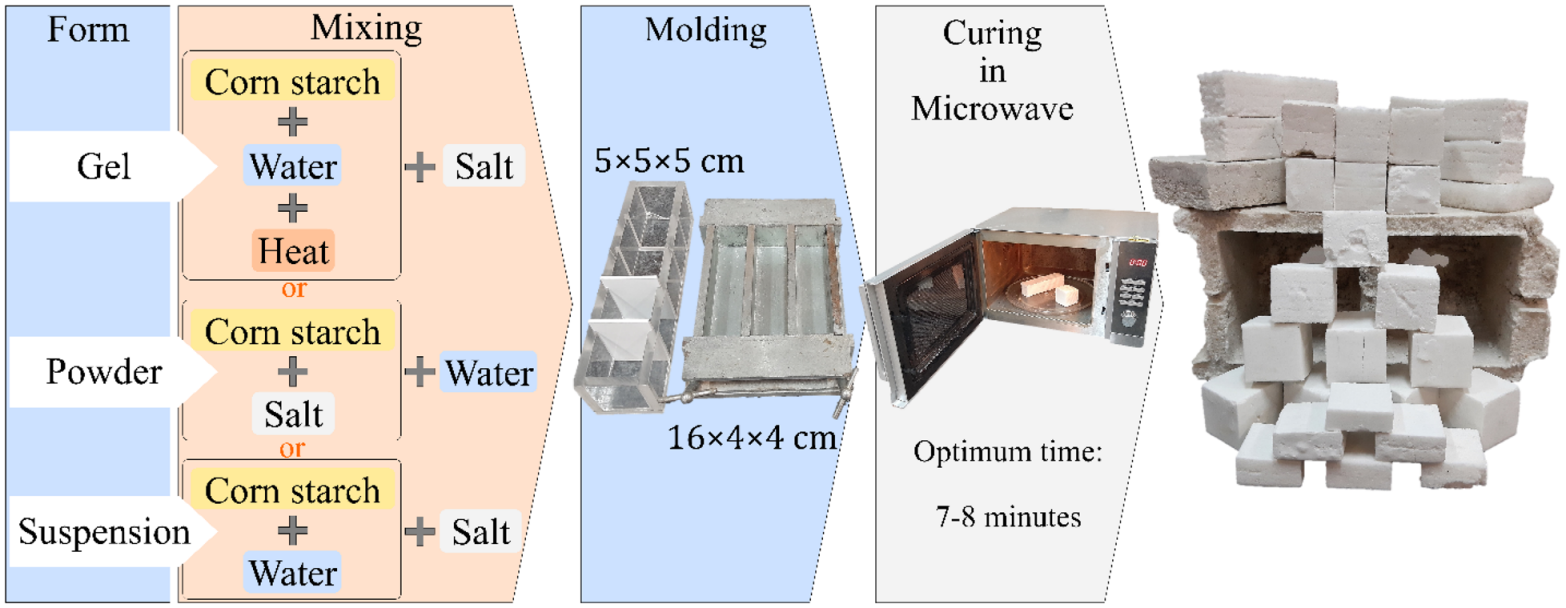
Overview of the manufacturing process, curing, and evaluation of mechanical strength of NaCl-binder samples.

An overview of the process of fabricating and curing NaCl-binder samples with and without starch and the evaluation of their mechanical strength are shown in Fig. 1. In this study, two types of salt, Type 1 and Type 2, were used for sample preparation.

### Hydrophobic NaCl-binder sample preparation method

The use of different materials to coat sand-starch samples was reported by Mansour et al., and according to the results, it was found that the use of paraffin wax and beeswax (a biodegradable natural material) leads to the formation of the highest contact angles^26^. As a result, in this study, samples of 1% starchy NaCl-binder were made using S2 salt, microwave cured for 7 min, and coated with paraffin wax and beeswax to assess their hydrophobic qualities for the first time. Then, the hydrophobic properties of the surface of the samples were qualitatively analyzed by measuring the contact angle of the samples with distilled water using the Jikan CAG-20 analyzer as a static contact angle analysis tool.

### Characterization of the NaCl-binder

To measure the mechanical strength of NaCl-binder samples, a concrete compression testing machine was used to measure the compressive strength of the samples by ASTM C39^50^. A flexure testing machine with a 3-point loading method by ASTM C348-21^51^ was used to measure flexural strength. The X-ray diffraction (XRD) analysis of the corn starch powder, 0% (wt.) starchy NaCl-binder powder and 1% (wt.) starchy NaCl-binder samples in the angle range (2θ) from 10–80° was also recorded with an Xpert Pro diffractometer (PW1730-PHILIPS, Netherlands) with a Cu anode at room temperature with a step size of 0.05°, 40 kV and 30 mA. To evaluate the chemical composition of the samples, in continue, salt (Type1 and Type2), corn starch powder, and 1% starchy NaCl-binder samples cured for 4 and 7 min in the microwave was determined by an X-ray fluorescence (XRF) spectrometer (PW1410-PHILIPS, The Netherlands). The target samples are exposed to X-rays during the XRF test, and secondary X-rays are generated as a result of the excitation of atoms. Afterward, the necessary element or elements can be found using Energy-dispersive X-ray spectroscopy (EDS). The microscopic morphology of the rock salt and 1% starchy NaCl-binder samples cured for 7 min were investigated at room temperature and 15 kV using MIRA3-FESEM (Tescan, Czech).

## Results and discussion

In this study, the production of NaCl-binder samples was done using extremely small amounts of starch (1 wt.%), especially in gel form, which is considered innovative. The issue of the samples' solubility in water is also resolved by coating the samples with paraffin wax and beeswax. In addition, desirable compressive strength was attained despite small corn starch consumption.

### Effect of preparation factors on compressive behavior of the NaCl-binder

#### The effects of using 1% starch and curing time on the behavior of the samples

**Figure 2:**
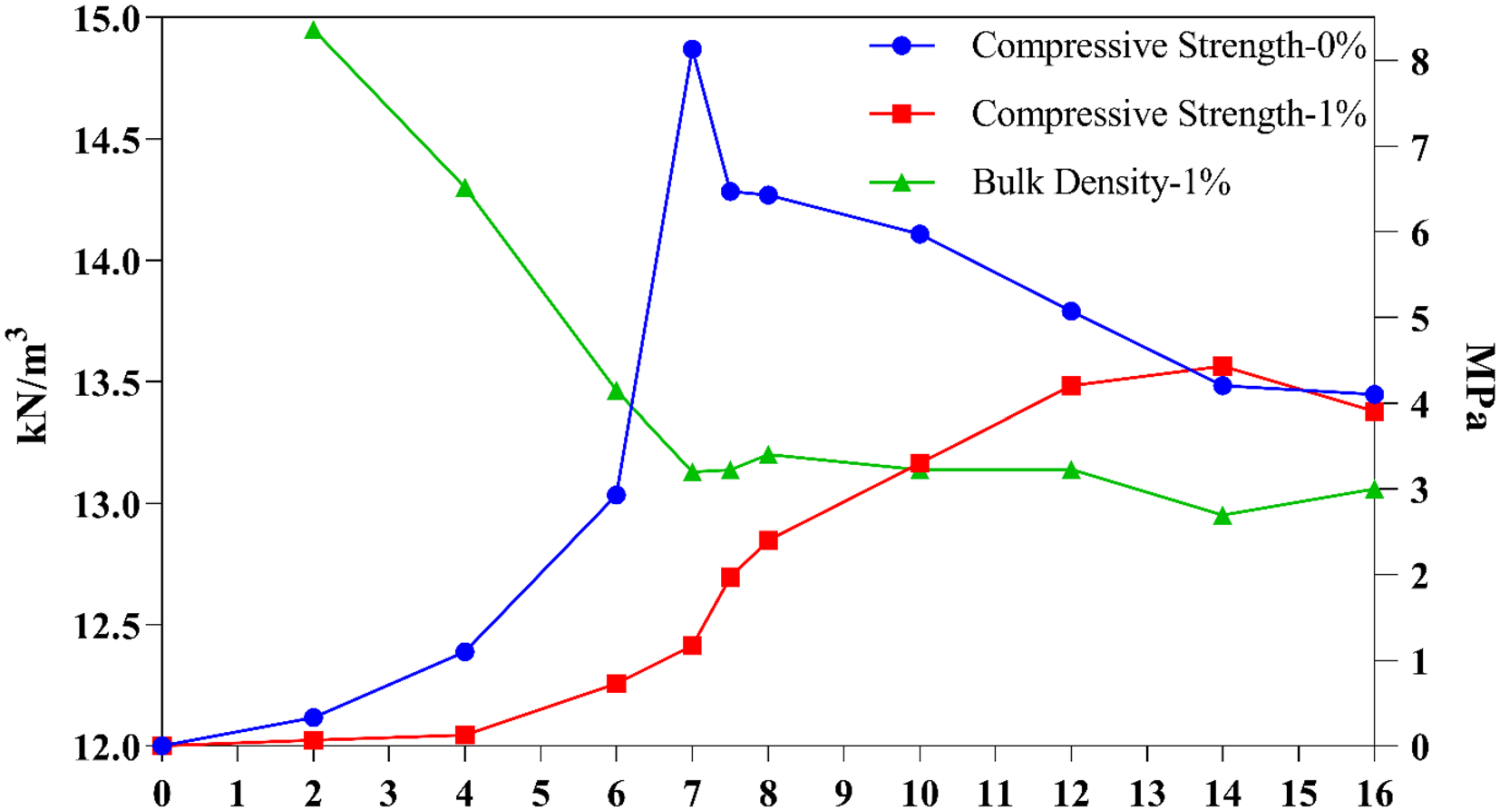
Effect of curing time on compressive strength, density, and color of NaCl-binder with 0% and 1% starch.

**Figure 3:**
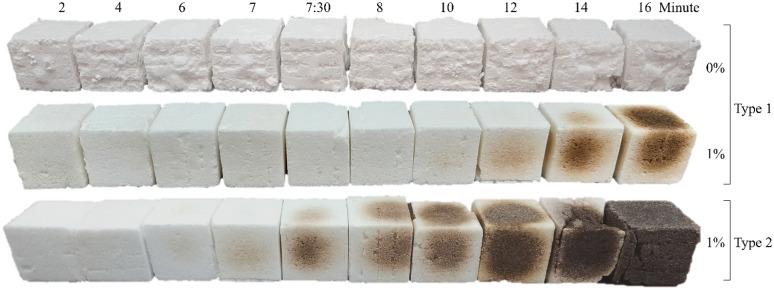
Color of NaCl-binder samples for different durations of microwave curing, different NaCl salt types with 0% and 1% corn starch.

In this part, the possibility of the economical production of NaCl-binder with low corn starch consumption (1 wt.%) with the lowest energy was evaluated. The histogram of the compressive strength of samples without starch and with 1% starch, cured in the microwave (along with the moving average diagram) is presented according to the diagram in Fig. 2. S2 salt with a particle diameter of 300–600 µm was used in the preparation of all of these samples. The average maximum 7-min compressive strength of NaCl-binder samples containing 1% starch is 8.13 MPa, while the maximum 7-min compressive strength of samples without starch is 1.16 MPa. As a first result, it is clear that when using 1% starch gel in the preparation of NaCl-binder samples, the compressive strength of the samples shows an average increase of about 600%. On the other hand, it can be seen from the diagram that the compressive strength of the samples containing 1% starch after reaching its peak value in 7 min of curing, with further increase of curing time, has taken a downward trend and for 14 to 16 min of curing time, it reaches about 4 MPa. On the other hand, the compressive strength of starch-free samples increases with increasing the curing time and reaches 4 MPa. According to the study by Kulshreshtha et al., since the burning temperature of corn starch is 290 °C, it was found that the starch in the sand starch samples (CoRncrete) started to burn with increasing curing time and the compressive strength of the samples decreased^20^. For the NaCl-binder samples with 1% starch, by increasing the curing time from 10 min, the starch is gradually burned, and its compressive strength tends towards the samples without starch, and the binding effect of the starch is lost. The color of samples without starch is white for different curing durations, while the color change of samples containing 1% starch with an increase in curing time, which leads to the burning of starch, is evident as shown in Fig. 3. According to Fig. 3, another important point is that by using 1% starch (in the form of a starch gel), the surface of NaCl-binder samples after microwave treatment is completely smooth, while the surface of starch-free samples after microwave treatment is quite non-smooth and uneven, which is important from the architectural aspect.

Figure 2 also shows the compressive strength and bulk density of 1% starchy NaCl-binder samples prepared using S2 salt. As is known, by increasing the curing time from 2 to 7 min, the compressive strength of the samples increases and reaches the maximum, and the bulk density of the samples decreases due to the evaporation of water. By increasing the curing time from 7 to 16 min, the compressive strength of the samples decreased and the bulk density of the samples remained constant at around 13 kN m^−3^, which is due to the complete evaporation of the physical water in around 7 min. By increasing the curing time, there is no physical water in the sample to evaporate and therefore the bulk density of the samples remains almost constant during the curing time of 7 to 16 min.

The compressive strength test results and Fig. 3 indicate that 7–8 min is the optimum period time for microwave curing to ensure the samples not only do not burn but also have a high compressive strength as well. Additionally, it is important to note that no prior study has been conducted on this issue, and these results are being presented for the first time.

**Figure 4:**
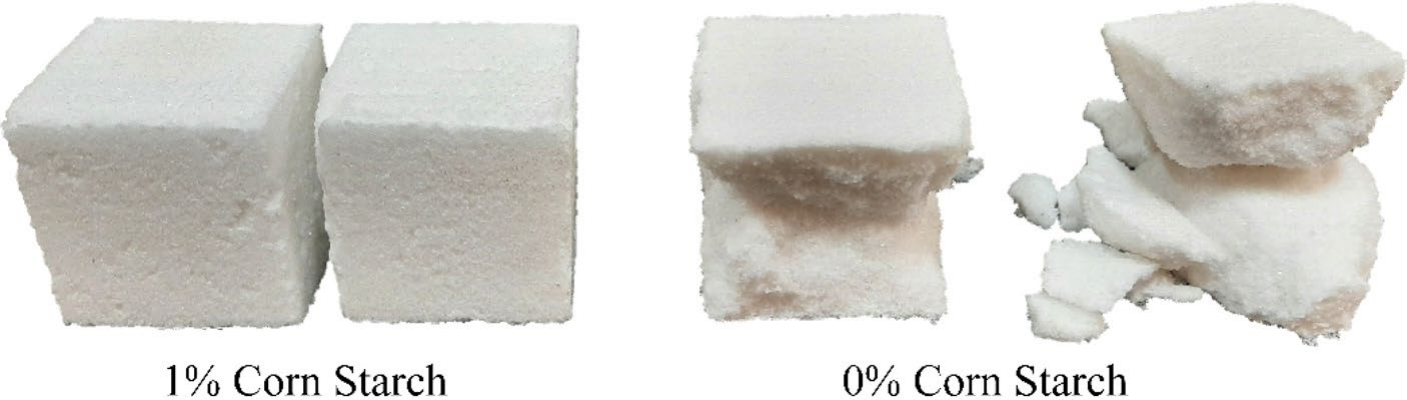
The effect of using 1% starch in NaCl-binder on sample fracture.

Figure 4 shows the fracture of 0 and 1% starchy NaCl-binder samples. The starch-free samples disintegrated after loading, while the 1% starch samples fully retained their cubic state. Due to the starch's adhesion, there were fewer surface cracks in the samples with 1% starch, indicating a soft fracture of these samples.

**Figure 5:**
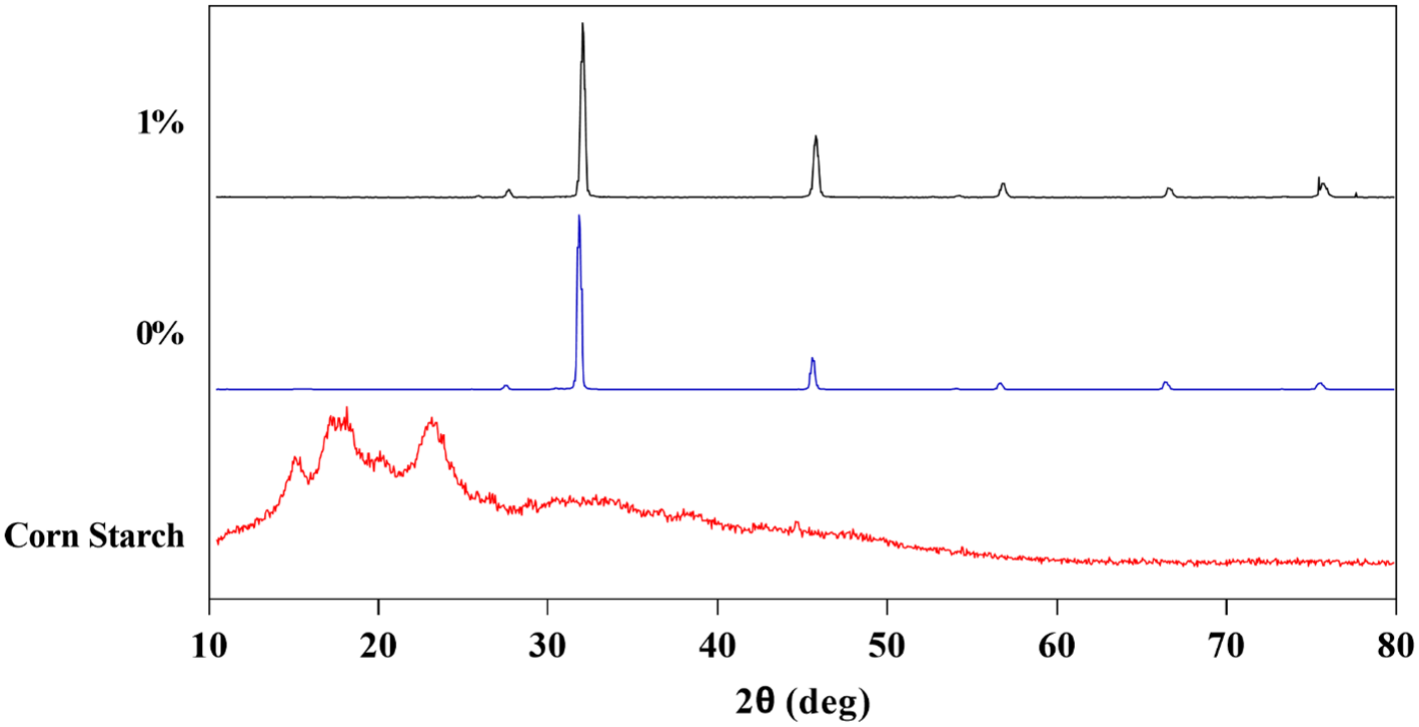
XRD diagram of 0 and 1% starchy NaCl-binder and corn starch samples.

**Figure 6:**
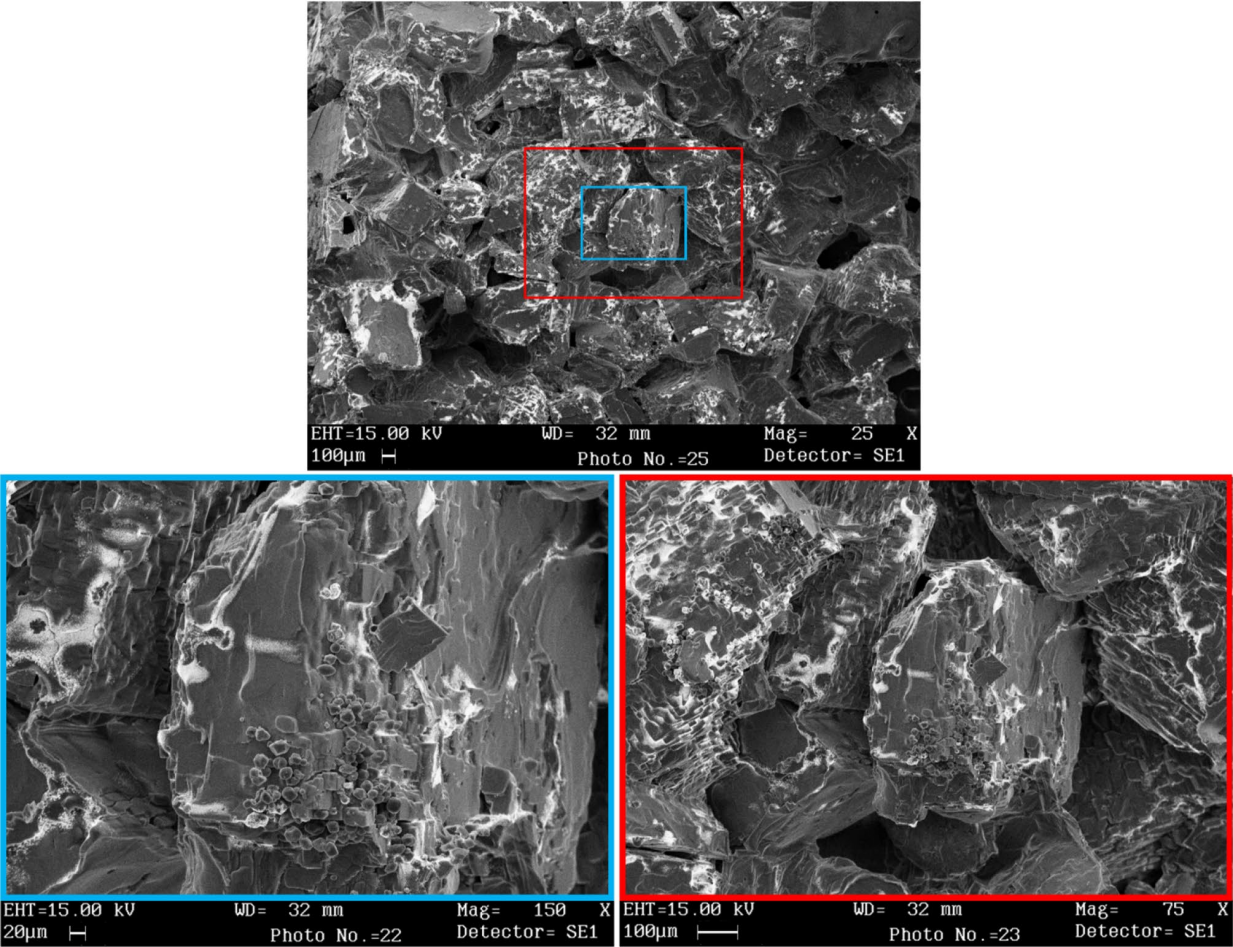
SEM images of 1% starchy NaCl-binder samples at ×25, ×75 and ×150 magnifications.

Table 3 shows the results of XRF analysis of 1% starchy NaCl-binder samples microwave cured for 4 and 7 min and the results of starch powder sample analysis. The LOI (loss on ignition) of starch powder is high due to its organic composition. The LOI for salt powder (type 1) is 4.07% and for 1% starchy NaCl-binder samples microwave cured for 4 min is 32.18%, which is due to the presence of starch gel (physical water and organic starch compound). But in the 1% starchy NaCl-binder that was microwave cured for 7 min, a significant portion of the water in the samples evaporated during curing, and therefore the LOI of this sample dropped to 11.12%. In salt powder and 1% starchy NaCl-binder samples, the amount of sodium element is about 38% and the amount of chlorine element is about 59% as the dominant elements. According to Fig. 5, the XRD diagram of 0 and 1% starchy NaCl-binder samples processed in the microwave for 7 min and a corn starch powder sample is presented. As it is known, due to the very low starch consumption in the NaCl-binder sample, the peaks of the two samples of 0% starchy NaCl-binder and 1% Starchy NaCl-binder are very similar and completely different from the peaks of the corn starch sample. Meanwhile, the strength of 1% starchy NaCl-binder samples is about 600% higher than that of 0% starchy NaCl-binder samples. The effect of using 1% starch to increase the strength of starch samples by 600% is one of the important results of the current research from a technical and economical point of view for the production of low-cost salt materials. To ascertain how salt components, bind to starch and each other, SEM analysis was performed to investigate the microstructure of NaCl-binder samples. According to Fig. 6, SEM images are shown at 25×, 50×, 75×, and 150× magnifications of 1% starchy NaCl-binder samples prepared with S2 salt (300–600 μm) and cured in the microwave for 7 min showed the layered support (starch gel-salt) consists of binding salt particles together. Of course, the empty space between the particles, which is caused by the evaporation of excess physical water, is also clearly visible.

**Table 3:** XRF analysis results of corn starch powder, rock salt powder, and 1% Starchy NaCl-binder samples cured for 4 and 7 min in the microwave.

Material	CaO	Na_2_O	K_2_O	TiO_2_	MnO	P_2_O_5_	Cl	Na	LOI*
Waste salt powder	0.586	–	0.039	0.019	0.007	N*	59.16	38.34	4.07
4 min-1% Starchy NaCl-binder	0.594	–	0.04	0.019	0.007	N	59.77	38.73	32.18
7 min-1% Starchy NaCl-binder	0.594	–	0.037	0.019	0.008	N	59.77	38.73	11.12

*LOI: Loss on ignition, N: not existed.

Furthermore, The results of this research differ significantly from those of other studies on salt materials, which supports the superior outcomes and innovation of the present study so that in the salt project related to Qatar, where the feasibility of making salty materials using salt extracted from seawater desalination using solar energy has been studied and mechanical strength and physical properties of the materials have been investigated^8^. In which, salt, maltodextrin, water, and alcohol were used, and the weight ratio of maltodextrin to salt was 12.5%, and the compressive strength and bulk density of the manufactured cubic samples were reported 2.99–7.57 MPa and 1.48–10.3 g cm^−3^, respectively. In the current research, by using S2 salt (300–600 μm), while removing alcohol from raw materials and substituting cheaper food-grade corn starch instead of maltodextrin, it is possible to make salt samples with a very low weight ratio of starch to salt of 1%. The average compressive strength and bulk density of the produced cubic samples were 8.13 MPa and 13.13 kN m^−3^ (1.34 g cm^−3^), respectively. Whereas, with increasing starch consumption, the compressive strength was also reached (discussed in the next parts). Another study presented a type of sustainable and new building material for use in hot arid climates called SaltBlock, which used raw materials such as ethylene–vinyl acetate polymer, salt powder, and fine sand to produce lightweight salt blocks^11^. In salt construction materials such as salt-rich mud mortar and salt mixed with natural resin, the mechanical strength and physical characteristics of the materials have not been evaluated^5–7^.

#### Granulation effect on the behavior of NaCl-binder

**Figure 7:**
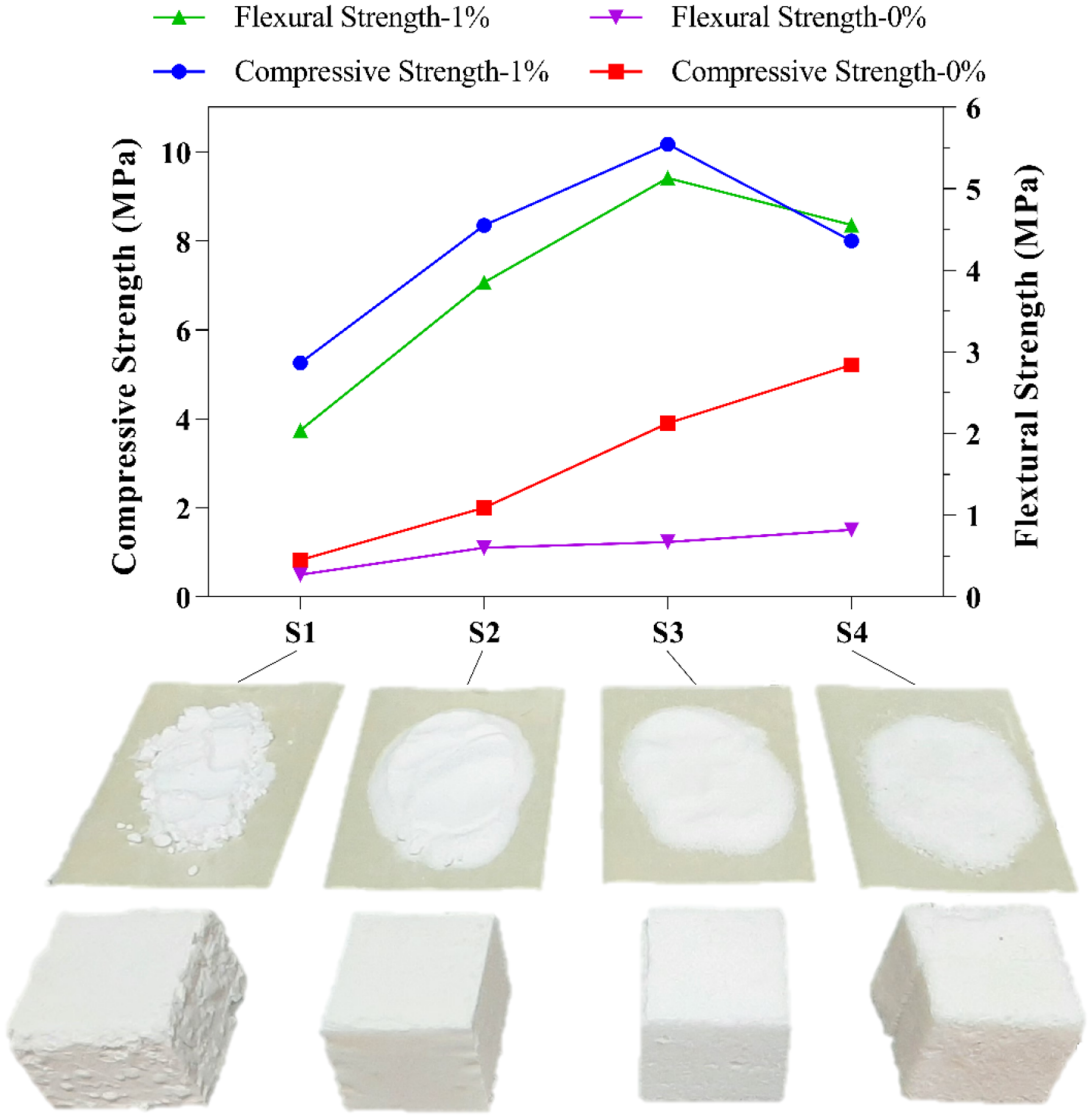
Compressive and flexural strength of NaCl-binder samples with different salt grain size.

**Figure 8:**
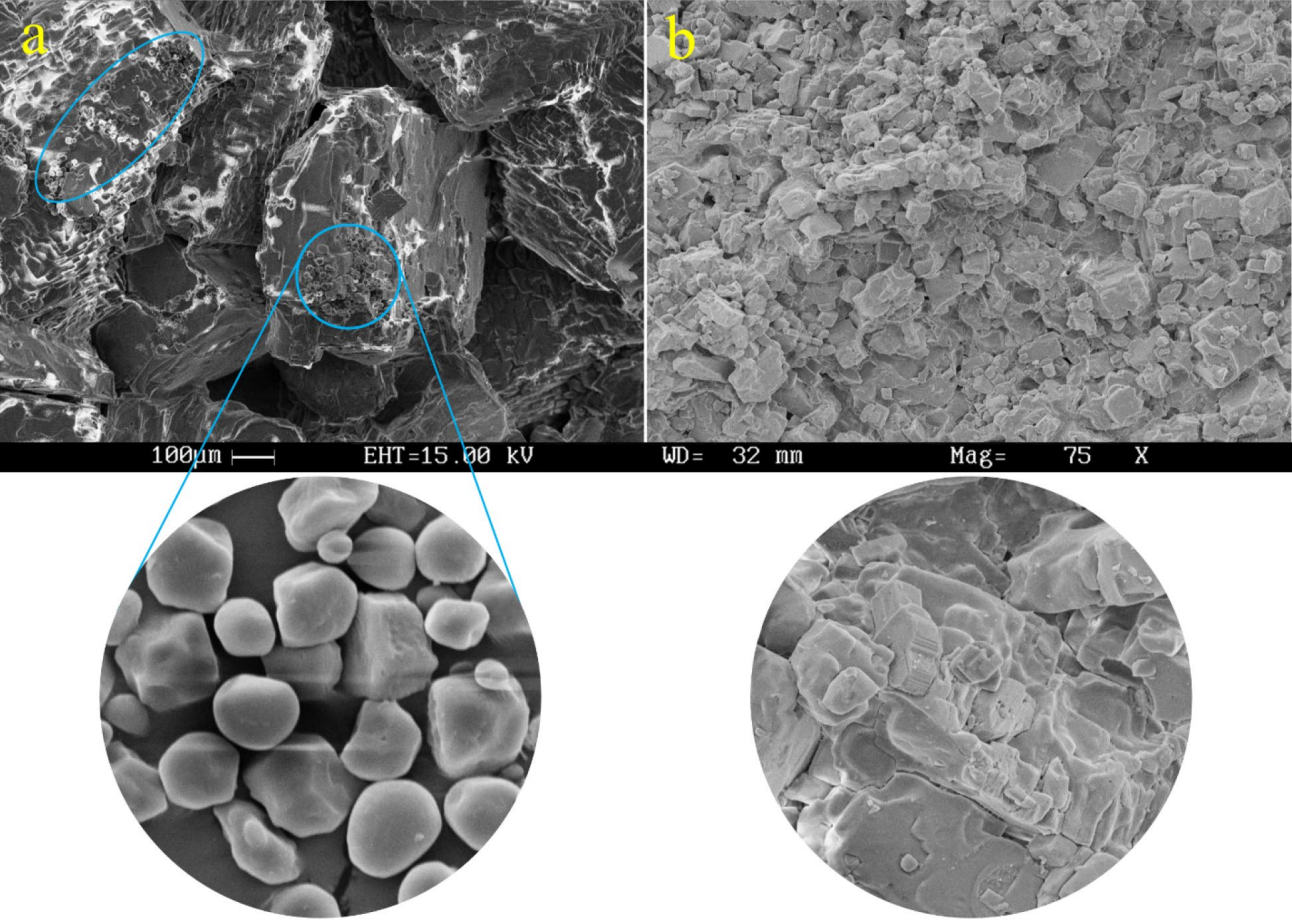
SEM images of 1% starchy NaCl-binder samples prepared with S2 (**a**) and S4 (**b**) at ×75 magnifications.

Aggregate grading is an essential key parameter of the compressive strength of different mortar samples. In the following section, samples with and without starch (0 and 1% starchy NaCl-binder) were prepared and tested with an optimal curing time of 7 min to investigate the impact of variations in salt grain size (S1, S2, S3, and S4) on compressive strength and flexural strength. According to Fig. 7, for salt particles with a diameter of < 0.15 mm (S4), 0.15–0.30 mm (S3), and 0.3–0.6 mm (S2), for samples with 1% starch, a compressive strength of about 8–10 MPa and a flexural strength of about 3–5 MPa were initially determined. The lowest compressive and flexural strengths were among the samples made using S4 salt (the largest particle diameter). Of course, it should be noted that samples with a larger particle diameter than S4 were also produced and their results were not presented in the form of graphs in this study due to the low compressive strength and very low flexural strength. Comparing the graphs in Fig. 7, it can be seen that the compressive strength of the samples made with 1% starch gel shows an increase from 54 to 533% compared to the samples without starch. The flexural strength of the samples made with 1% starch gel also shows a significant increase from 456 to 665% compared to samples without starch. In this way, the effect of using 1% starch to increase the mechanical strength of the samples can be clearly seen. Referring to Fig. 8, the corresponding SEM images of 1% starchy NaCl-binder samples prepared with Salt S2 (300–600 μm) and S4 (< 150 μm) are presented. Greater flexural strength was obtained in the sample made with finer particles due to the larger surface area of the interconnected particles.

As a result, S3 is the most suitable salt size out of the various salt sizes, and samples from S1, S2, and S4 all fall into the same strength range. Furthermore, according to Fig. 7, due to the nearly identical compressive strength and flexural strength values for S2 and S4 for 1% starchy NaCl-binder samples, the SEM images of these two samples were examined to determine the reason for the lack of change in mechanical strength (very slight change) caused by changes in salt size. In light of this, it is concluded that the majority of the mechanical strength is provided by the salt particles. If the salt particles are very small, some of the mechanical strength is transferred to the small starch particles (with smaller strength). However, unlike the excellent adhesion between fine salt particles and starch particles (Fig. 8b), the salt particles do not have enough ability to bear the force, and as a result, the strength decreases. Additionally, if large salt particles are used, the strength will decrease because of the imbalance in the granulation size between the particles, which prevents the large salt particles from connecting to each other properly and wastes a significant amount of starch particles on their unconnected surfaces (Fig. 8a).

In addition to the above, studies on CoRncrete and starch-sandstone have confirmed the same issue. Because the use of 1% starch is insufficient to bind the sand particles together, much higher amounts of starch were used in the studies to achieve the desired mechanical strength^25,26^. Additionally, unlike sand particles, salt particles of NaCl-binder can bind to one another in the presence of water and provide a favorable strength.

#### The effect of using higher amounts of starch consumption

As mentioned, in the salt project related to Qatar, salt, maltodextrin, water, and alcohol were used, and the weight ratio of maltodextrin to salt was 12.5%, and the compressive strength and bulk density of the manufactured cubic samples were reported 2.99–7.57 MPa and 1.48–10.3 g cm^−3^, respectively. In the current research, by removing alcohol from raw materials and substituting cheaper food-grade corn starch instead of maltodextrin, and using a very low weight ratio of starch to salt of 1%, NaCl-binder material is introduced. In the following, the effect of using *higher amounts of starch was evaluated.*

**Figure 9:**
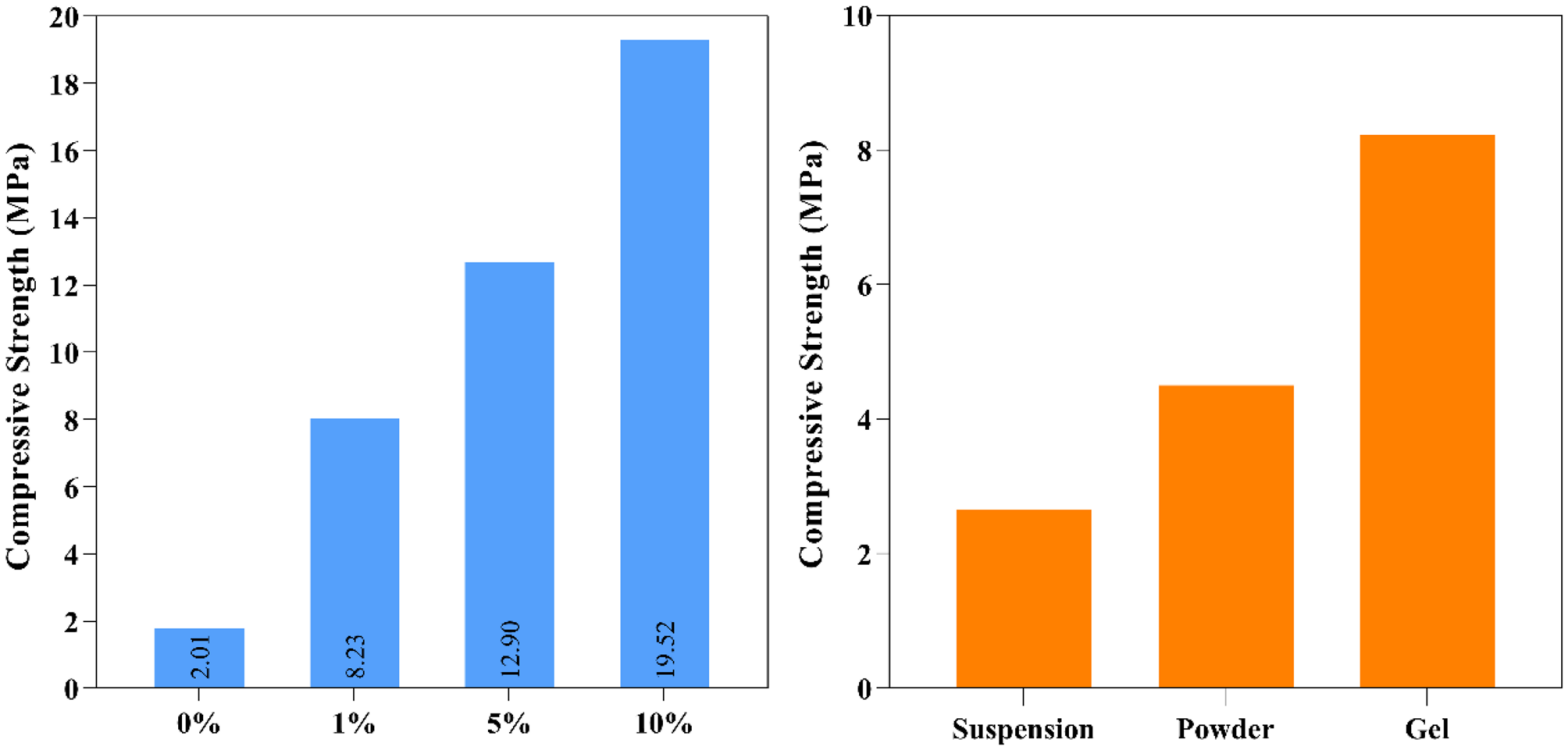
Comparison of the compressive strength of samples with different forms of starch (right) and samples with different amounts of starch (left).

The compressive strength of samples made with different amounts of starch (0, 1, 5, and 10%) is shown in Fig. 9 left. By increasing the starch consumption from 0 to 1%, the compressive strength of the samples increases by about 600%. Increasing starch consumption from 1 to 10% increases compressive strength by about 137% (increase from 8.23 to 19.52 MPa).

The compressive strength of the NaCl-binder samples can be addressed in relation to the findings of the clay brick (unfired) test and the corresponding standards^52^. The NaCl-binder samples with 1% and 5% corn starch conform to Grade NW and samples with 10% corn starch conform to Grade SW according to ASTM C62-17 in terms of load capacity^53^.

#### The effect of starch form on the compressive strength of samples

An experiment using three different mix designs were conducted to investigate the impact of the production methods on the compressive strength of the NaCl-binder (for further details, see Fig. 1). Figure 9 right compares the compressive strength of samples that have been microwave-cured for 7 min and made with different starch forms including powder, suspension, and starch gel. In all these cases the starch is 1% by weight and the highest compressive strength is related to the sample made with starch gel. The lowest compressive strength of the samples is related to the use of 1% starch in suspension. The reason lies in the impossibility of a more uniform distribution of the starch particles in the suspension between the salt particles compared to the starch gel and in the lower viscosity of the starch suspension compared to the starch gel.

#### Effect of the NaCl salt type

Due to the wide range of salt sources that can be used to make NaCl-binder, two NaCl salt types were selected in order to compare the samples that were obtained from each of them. Table 4 shows the results of the XRF analysis of two NaCl salt types (type 1 and type 2) for the preparation of 1% starchy NaCl-binder samples. Figure 3 also shows samples of 1% starchy NaCl-binder made using two types of salt and microwave cured for 2 to 16 min, showing the effect of using type 2 salt with a higher LOI (amount of water and organic impurities). In which, burnt samples during microwave treatment are clearly recognizable. Therefore, the type of NaCl salt as a raw material is very important.

**Table 4:** XRF analysis results of corn starch powder, rock salt powder, and 1% Starchy NaCl-binder samples cured for 4 and 7 min in the microwave.

Material	CaO	Na_2_O	K_2_O	TiO_2_	MnO	P_2_O_5_	Cl	Na	LOI*
Salt powder-type 1	0.586	–	0.039	0.019	0.007	N*	59.16	38.34	4.07
Salt powder-type 2	0.336	–	N	0.018	0.007	0.005	59.95	38.84	28.04
4 min-1% Starchy NaCl-binder**	0.594	–	0.04	0.019	0.007	N	59.77	38.73	32.18
7 min-1% Starchy NaCl-binder**	0.594	–	0.037	0.019	0.008	N	59.77	38.73	11.12
4 min-1% Starchy NaCl-binder***	0.341	–	N	0.018	0.007	0.005	60.03	38.90	45.21
7 min-1% Starchy NaCl-binder***	0.340	–	N	0.018	0.007	0.005	60.03	38.90	16.19

*LOI: Loss on ignition, N: not existed.

**Prepared by Salt Powder-type 1.

***Prepared by Salt Powder-type 2.

### Hybrid ecofriendly-based coating

**Figure 10:**
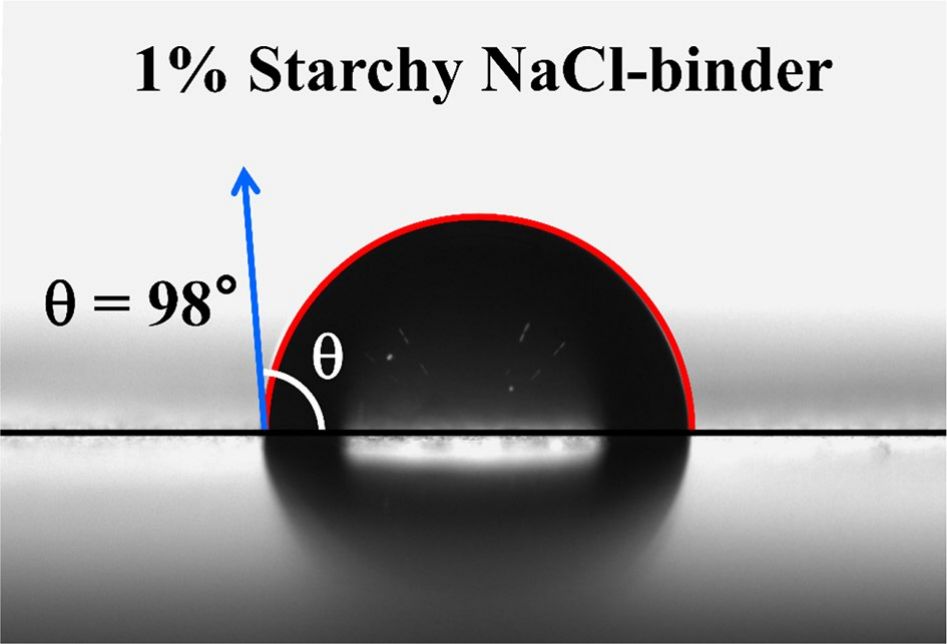
The effect of paraffin wax and beeswax on the hydrophobicity of a 1% starchy NaCl-binder sample.

Because salt is easily dissolved in water, products built with salt have a great potential for water solubility. Therefore, it is necessary to use hydrophobic materials for the coating of starchy NaCl-binder samples to use them as construction and load-bearing materials. Additionally, if the coating materials be biodegradable, they have better compatibility with the environment and cause less environmental harm. Among the starch sandstone samples coated with various biodegradable natural materials, the samples coated with paraffin wax and beeswax showed the most hydrophobic properties^26^. In this part, this hybrid ecofriendly-based material was examined for NaCl-binder coating. As shown in Fig. 10, a contact angle of 98° was achieved by coating the 1% starchy NaCl-binder samples with paraffin wax and beeswax. The contact angle is a technical index used to determine the hydrophobic properties of materials. In general, solid surfaces with contact angles (CA) less than 90° are considered hydrophilic and those greater than 90 are hydrophobic^54,55^.

It is claimed that NaCl-binder is entirely hydrophobic for the first time and may be utilized in structures in the presence of moisture based on the acquired contact angle, making NaCl-binder a material with tremendous potential in the construction sector.

## Conclusions

In this research, a new lightweight building material called starchy NaCl-binder was introduced, containing salt particles of different granulations as the main matrix material, starch as a co-binder and water. The reason for using the term co-binder is that, unlike sand particles, salt particles can bind together, and starch plays the role of a supplementary binder in the better binding of salt particles.

The important results of this research include the following:

- Introduction of a new lightweight and cost-effective material, 1% starchy NaCl-binder with a salt matrix and a particle diameter of 300–600 µm and a corn starch gel glue with a compressive strength of 8.13 MPa, a flexural strength of 3.85 MPa and a bulk density of 1.34 g cm^−3^
- Innovative use of starch gel rather than starch powder to produce 1% starchy NaCl-binder samples for homogeneous distribution of the supplementary binder on the salt particles.
- Manufacturing of 1% starchy NaCl-binder materials with different grain sizes of salt particles and obtaining a maximum compressive strength of 10.17 MPa and a flexural strength of 5.13 MPa
- Ability to produce 0% starchy NaCl-binder samples without using starch and only salt particles with a compressive strength of 5.21 MPa and a flexural strength of 0.82 MPa (significant reduction in flexural strength when no starch is used)
- Obtaining the optimal curing time of 7 min in the microwave to produce 1% starchy NaCl-binder samples with maximum compressive strength and white color without burning.
- Achieving the optimal curing time of 14 min in the microwave to produce 0% starchy NaCl-binder samples with the maximum compressive strength possible.
- Manufacturing 10% starchy NaCl-binder materials with salt matrix and co-binder of corn starch with a higher compressive strength of 19.52 MPa and hence higher cost by using more starch
- Successful hydrophobization of samples made with hybrid ecofriendly-based material, a mixture of paraffin wax and beeswax.

These new materials can be a candidate for the materials of salt buildings and other structures along with increasing tourism. By using the salt obtained from the desalination of seawater using solar energy, it can be used to produce salt materials, which have dual environmental benefits. On the one hand, eco-compatible salt materials can be used for the construction of salt buildings and tourism development without the use of polluting materials and types of cement, and on the other hand, by preventing the discharge of separated salt water to the sea, related environmental problems are solved.

## Data Availability

The datasets used and analyzed during the current study available from the corresponding author on reasonable request.
